# Which Parameters of Beat-to-Beat Blood Pressure Best Predict Poor In-Hospital Outcome in Spontaneous Intracerebral Hemorrhage?

**DOI:** 10.3389/fnagi.2020.603340

**Published:** 2020-11-19

**Authors:** Zhen-Ni Guo, Yang Qu, Hailili Reziya, Jia Liu, Xiu-Li Yan, Peng Zhang, Pan-Deng Zhang, Shuang Qi, Yi Yang

**Affiliations:** ^1^Department of Neurology, Stroke Center & Clinical Trial and Research Center for Stroke, The First Hospital of Jilin University, Changchun, China; ^2^China National Comprehensive Stroke Center, Changchun, China; ^3^Jilin Provincial Key Laboratory of Cerebrovascular Disease, Changchun, China; ^4^Shenzhen Institutes of Advanced Technology, Chinese Academy of Sciences, Shenzhen, China

**Keywords:** intracerebral hemorrhage, blood pressure variability, blood pressure, outcome, stroke

## Abstract

**Objective:** There is increasing evidence that high blood pressure (BP) levels and BP variability (BPV) over 24 h or longer are associated with poor clinical outcomes in patients with intracerebral hemorrhage (ICH). The objective of this study was to examine the association between different beat-to-beat BP parameters and in-hospital outcomes.

**Methods:** Patients with a diagnosis of acute spontaneous ICH were recruited consecutively and prospectively between September 2018 and January 2019. Beat-to-beat recordings were measured non-invasively for 5 min within the first 72 h after the onset of symptoms. BPV was analyzed by standard deviation (SD), coefficient of variation (CV), average real variability (ARV), and variation independent of mean (VIM). Outcome was assessed at discharge using the modified Rankin Scale (mRS) score. Multivariate logistic regression analysis was used to assess the association between BP levels, BPV, and clinical outcomes.

**Results:** A total of 66 patients were included, of whom 34 had poor outcomes (mRS score, 3–6). Patients with poor outcomes had significantly higher National Institute of Health Stroke Scale scores (4.5 vs. 9, *p* < 0.001), a larger ICH volume (8 vs. 14.5 mL, *p* = 0.004), and an increased systolic BP (SBP) -CV (3.2 vs. 4.8, *p* < 0.001) and diastolic BP (DBP) -CV (3.7 vs. 4.9, *p* = 0.015). After adjustment for major covariates, multivariate logistic regression analysis revealed that SBP-CV was independently associated with an increased risk of poor in-hospital outcomes [odds ratio (OR) 2.535; 95% confidence interval (CI), 1.211–5.305; *p* = 0.014]. The receiver operating characteristic area for SBP-CV in predicting poor in-hospital outcome was 0.827 (95% CI, 0.730–0.925; *p* < 0.001), and the best cutoff point was 3.551 (sensitivity, 82.35%; specificity, 68.75%).

**Conclusion:** A higher beat-to-beat BPV in the first 72 h of admission was associated with unfavorable in-hospital outcomes in patients with ICH. The stabilization of BPV during the acute phase may be a therapeutic target; this could be tested in future clinical trials.

## Introduction

Spontaneous intracerebral hemorrhage (ICH) is a major cause of disability and mortality among patients with different types of stroke, and few effective treatment options are available (Flaherty et al., 2006). Elevated blood pressure (BP) is a risk factor for both ICH and worse clinical outcome (Qureshi et al., 2016). However, a meta-analysis of five randomized, controlled, single/double-blinded, parallel trials found no differences between aggressive and conservative BP-lowering strategies in the incidence rates of 3-months mortality and early neurological deterioration (Lattanzi et al., 2017). Post hoc analyses of the Field Administration of Stroke Therapy-Magnesium trial, Intensive Blood Pressure Reduction in Acute Cerebral Hemorrhage Trial, and Antihypertensive Treatment of Acute Cerebral Hemorrhage II trial showed that increased BP variability (BPV) was associated with a poor functional outcome (Manning et al., 2014; Chung et al., 2018; de Havenon et al., 2018). This indicated that, in addition to the absolute BP level, BPV might also be a significant risk factor for worse outcomes after ICH.

Beat-to-beat BPV is considered to be a very short-term BPV. In ischemic stroke, beat-to-beat BPV has been found to be associated with worse clinical outcomes, and might be associated with an increased risk of recurrent events (Dawson et al., 2000; Webb et al., 2018). Beat-to-beat BP parameters can be obtained continuously, non-invasively and have been correlated with baroreceptor sensitivity and autonomic nervous systematic function (Parati et al., 2013; Kishi, 2018). Previous studies have indicated that ICH is followed by an increase in sympathetic nervous system activity, as evidenced by increased plasma catecholamine and corticosteroid levels, and damage to the baroreceptor reflex arc (Meyer et al., 1973; Feibel et al., 1981). However, the characteristics of beat-to-beat BP parameters in acute ICH remain unclear. To date, no study has systematically evaluated the role of beat-to-beat systolic BP (SBP), diastolic BP (DBP), SBPV, and DBPV with clinical outcomes in acute ICH.

Hence, the main objective of this study was to identify any potential prognostic differences between the various parameters of beat-to-beat BP in outcome in patients with acute spontaneous supratentorial ICH.

## Materials and Methods

### Participants

Patients with acute spontaneous supratentorial ICH who had been admitted to our stroke unit within the past 72 h were recruited consecutively and prospectively between September 2018 and January 2019. The diagnosis of ICH was based on computed tomography (CT, 64-slice, Somatom Definition; Siemens Healthcare, Germany) of the brain. Demographic data (age and sex), medical history, and vascular risk factors, including alcohol consumption, cigarette smoking, hypertension, diabetes mellitus, previous coronary heart disease, ischemic stroke, and ICH, were documented. Antihypertensive and diabetic medications were defined as taking any drugs intravenously or orally before or after admission. The initial stroke severity was assessed using the National Institute of Health Stroke Scale (NIHSS) upon admission to the stroke unit. The primary locations of ICH were classified into the basal ganglia, thalamic, or lobar areas. The presence of intraventricular hemorrhage was also recorded. The ICH volume was calculated from the first CT scan using the (*a* × *b* × *c*)/2 method, where *a* is the greatest hemorrhage diameter by CT, *b* is the diameter 90° to *a*, and *c* is the approximate number of CT slices with hemorrhage multiplied by the slice thickness (Kothari et al., 1996; Huttner et al., 2006). Although hospitalized, 43 of 66 subjects underwent an additional 1 or 2 CT scans according to their clinical status, as determined by a neurologist at different time points. We did not use those data because of a potential selection bias. All patients received standard medical treatment according to the current guidelines from the American Heart Association/American Stroke Association in the comprehensive stroke center of our hospital (Hemphill et al., 2015).

The exclusion criteria of this study were as follows: age <18 or >80 years; premorbid modified Rankin Scale (mRS) ≥2; patients who were unable to cooperate sufficiently to complete the beat-to-beat monitoring (for example, due to conditions such as atrial fibrillation during the recording).

This study was approved by the Ethics Review Committee of the First Hospital of Jilin University, and written informed consent was obtained from all participants or their direct relatives.

### BP Recording and Variability Measures

Admission BP was measured at the brachial artery using an automatic BP monitor (Omron 711, Japan). Beat-to-beat recordings of SBP (in mmHg) and DBP (in mmHg) were measured noninvasively for 5 min within the first 72 h after the onset of symptoms using a servo-controlled plethysmograph (Finometer model 1, FMS, Rotterdam, the Netherlands) on the middle finger in a specific, quiet examination room with a controlled temperature ranging from 20 to 24°C. Because BPV was heterogeneous according to different measuring time points, all beat-to-beat BP was recorded 9:00 to 10:00 AM. Before the examination, all participants were asked to relax in a supine position for 10 min in the room.

BPV on beat-to-beat monitoring was calculated for 5 min. Continuous recordings of beat-to-beat information were processed by MATLAB (R2017b, MathWorks, USA) using scripts developed by the research team. Ectopic beats and artifacts were automatically detected, visually reviewed, and removed by linear interpolation (Webb et al., 2018). Linear interpolation was conducted using the routine function “interp1” provided by MATLAB. Mean SBP and DBP were defined as the average BP measurements. Beat-to-beat BPV, including SBPV and DBPV, was evaluated using the standard deviation (SD), coefficient of variation (CV), average real variability (ARV), and variation independent of mean (VIM) of BP measurements. The SD and CV were the most frequently used measuring values in previous studies (Xia et al., 2017). However, the SD does not consider the time sequence of individual BP measurements, and CV might be correlated with the mean BP. On that basis, ARV and VIM were calculated (Mena et al., 2005; Rothwell et al., 2010; Xia et al., 2017). The formulas and characteristics of these parameters were shown in Table 1.

**Table 1:** The formulas and characteristics of different blood pressure variability variables.

**Variables**	**Units**	**Formulas**	**Characteristics**
Standard Deviation (SD)	mmHg	$SD=\sqrt{\frac{1}{n-1}\overset{n}{\underset{i=1}{\sum }}{(X_{i}-X)}^{2}}$	Only reflecting the global fluctuation of BP measurements around the mean value; not taking the time sequence of measurements into account
Coefficient of Variation (CV)	nu.	$CV=100\frac{SD}{X}$	Correlating with the mean BP
Average Real Variability (ARV)	mmHg	$ARV=\frac{1}{n-1}\overset{n}{\underset{i=1}{\sum }}|X_{i+1}-X_{i}|$	Taking the time series variability into account
Variation Independent of Mean (VIM)	nu.	$VIM=kSD/{\bar{X}}^{m}$	Eliminating the effects of mean BP levels

*n is the number of BP measurements of a subject; X1, X2, …, Xn denotes a set of BP measurements, $\bar{X}$ represents the mean value of the set of BP measurements, k and m were obtained from a fitting curve of the form y = kx^m^ through a plot of SD BP (y-axis) against mean BP (x-axis), the parameter m is estimated from the data and k is a constant which can be chosen such that the values of VIM are on the same scale as values of SD*.

*BP, Blood Pressure*.

### Outcome

The clinical outcome was assessed at discharge using the mRS, with mRS scores ranging from 0 (no symptoms) to 6 (death). We defined mRS scores ≥3 as a poor outcome and mRS scores ≤2 as a favorable outcome.

### Statistical Analysis

Statistical Program for Social Sciences version 22.0 (SPSS; IBM, West Grove, PA) was used for statistical analysis. The distribution of continuous variables was assessed using a one-sample Kolmogorov-Smirnov test. Normally distributed data are presented as the means and SD, and non-normally distributed data are presented as the median and interquartile range. The clinical characteristics and BPV parameters used in this study were dichotomized into favorable and poor outcome groups. Intergroup differences were compared using the Student's *t*-test or Mann–Whitney test for continuous variables, and the chi-squared test or Fisher's exact test for categorical variables. The variables with a univariate between-group comparison with a *p* < 0.1 were eligible for inclusion in the multivariate logistic regression models as the major covariates. Multivariate logistic analysis was conducted to explore the association between BPV and clinical outcome, and odds ratios (ORs) and 95% confidence intervals (CIs) were used to evaluate the risk of poor outcomes. A receiver operating characteristic (ROC) curve of the statistically significant variables associated with poor in-hospital outcome was drawn. All statistical tests were two-tailed, and *p* < 0.05 was considered statistically significant.

## Results

Altogether, 66 patients with acute ICH were enrolled in the study, including 34 (51.5%) with poor outcomes (mRS score 3–6) and 32 (48.5%) with favorable outcomes (mRS score 0–2) at discharge. The overall characteristics of participants are shown in Table 2. The mean age of patients was 54.4 ± 10.1 years and 81.8% were men. The median NIHSS score at admission was 6 (interquartile range, 4–9), the median hemorrhage volume was 11.5 mL (interquartile range, 6–11.2 mL), and 18.2% had intraventricular hemorrhage. The average length ± SD of patient hospitalization was 12 days (interquartile range, 10.8–14.3 days).

**Table 2:** Comparison of demographic and clinical characteristics between patients with favorable (mRS, 0–2) and poor (mRS, 3–6) outcomes.

**Variables**	**Total (*n* = 66)**	**Favorable outcome (*n* = 32)**	**Poor outcome (*n* = 34)**	***P*-value**
Age (year)	54.4 ± 10.1	53.9 ± 11.1	54.9 ± 9.1	0.698
Sex (male, *n* [%])	54 (81.8%)	25 (80.6%)	29 (82.9%)	0.450
Cigarette smoking, *n* (%)	28 (42.4%)	12 (38.8%)	16 (45.7%)	0.432
Alcohol consumption, *n* (%)	27 (40.9%)	11 (35.5%)	16 (45.7%)	0.295
Coronary heart disease, *n* (%)	8 (12.1%)	5 (16.1%)	3 (8.6%)	0.397
Hypertension, *n* (%)	63 (95.5%)	30 (96.8%)	33 (94.3%)	0.519
Diabetes mellitus, *n* (%)	6 (9.1%)	4 (12.9%)	2 (5.7%)	0.350
Previous ischemic stroke, *n* (%)	9 (13.6%)	4 (12.9%)	5 (14.3%)	0.794
Previous intracerebral hemorrhage, *n* (%)	8 (12.1%)	4 (12.9%)	4 (11.4%)	0.927
Antihypertensive medication, *n* (%)	52 (78.8%)	26 (83.9%)	26 (74.2%)	0.281
Diabetic medication,anitha *n* (%)	4 (6.1%)	2 (6.5%)	2 (5.7%)	0.950
Admission NIHSS score	6 (4–9)	4.5 (3–6)	9 (6–11)	<0.001
Hospitalization length	12 (10.8–14.3)	12 (10.3–14)	13 (10.8–15)	0.518
**Location**				
Basal ganglia, *n* (%)	53 (80.3%)	25 (80.6%)	28 (80.0%)	0.666
Thalamus, *n* (%)	10 (15.1%)	6 (19.4%)	4 (11.4%)	0.629
Lobar, *n* (%)	3 (4.5%)	1 (3.2%)	2 (5.7%)	0.591
ICH volume (mL)	11.5 (6–11.2)	8 (4.3–14.3)	14.5 (10–18)	0.004
Presence of IVH, *n* (%)	12 (18.2%)	4 (12.9%)	8 (22.9%)	0.246
Admission SBP (mmHg)	165.6 ± 19.4	164.1 ± 17.1	167.2 ± 21.9	0.526
Admission DBP (mmHg)	97.3 ± 14.3	94.3 ± 12.7	100.5 ± 15.3	0.180
Mean SBP (mmHg)	147.6 ± 21.4	144.1 ± 21.1	151.3 ± 21.5	0.175
Mean DBP (mmHg)	80.2 ± 16.6	78.7 ± 16.9	81.8 ± 16.3	0.439
SBP-SD (mmHg)	5.8 ± 2.5	5.6 ± 1.9	6.1 ± 2.9	0.388
DBP-SD (mmHg)	3.3 ± 1.5	3.2 ± 1.2	3.5 ± 1.6	0.378
SBP-CV	4.0 ± 1.6	3.2 ± 0.9	4.8 ± 1.7	<0.001
DBP-CV	4.3 ± 2.1	3.7 ± 1.4	4.9 ± 2.4	0.015
SBP-ARV (mmHg)	2.5 ± 1.5	2.5 ± 1.3	2.5 ± 1.7	0.881
DBP-ARV (mmHg)	1.2 ± 0.5	1.2 ± 0.5	1.2 ± 0.5	0.909
SBP-VIM	5.9 ± 2.4	5.6 ± 1.9	6.2 ± 2.8	0.333
DBP-VIM	3.4 ± 1.5	3.2 ± 1.2	3.5 ± 1.7	0.322

*Data are expressed as mean ± standard deviation or n (%), except admission NIHSS score, hospitalization length, and ICH volume are median (interquartile range)*.

*NIHSS, National Institutes of Health Stroke Scale; ICH, intracerebral hemorrhage; IVH, intraventricular hemorrhage; SBP, Systolic Blood Pressure; DBP, Diastolic Blood Pressure; SD, Standard Deviation; CV, Coefficient of Variation; ARV, Average Real Variability; VIM, Variation Independent of Mean*.

A comparison of the patient characteristics between the favorable and poor groups are presented in Table 2. There were no differences in age, sex, vascular risk factors, or antihypertensive and diabetic medication between the poor outcome and favorable outcome groups. However, there was a significantly different NIHSS score at admission (4.5 vs. 9, *p* < 0.001) and ICH volume (8 vs. 14.5 mL, *p* = 0.004) between the two groups. As for BP magnitude, the poor outcome group had a tendency toward higher admission BP and mean BP than the good outcome group. For beat-to-beat BPV, poor outcome patients had an increased SBP-CV (3.2 vs. 4.8, *p* < 0.001) and DBP-CV (3.7 vs. 4.9, *p* = 0.015) and the other parameters also tended to be higher, although this was not significant.

**Figure 1:**
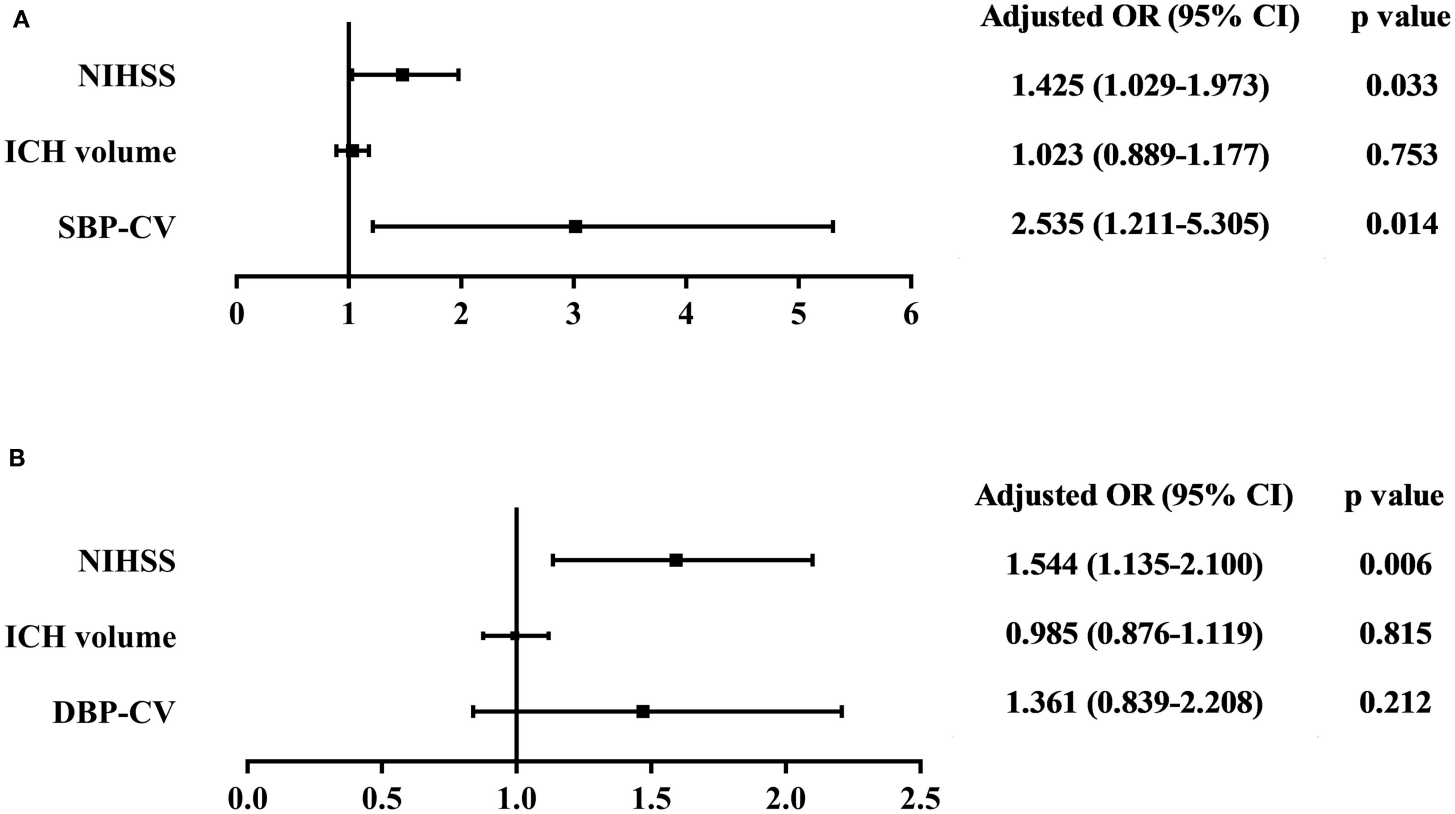
Association between **(A)** SBP-CV, and **(B)** DBP-CV and poor in-hospital outcome. The independent variables included in the multivariate logistic regression analysis were admission NIHSS score, ICH volume, and beat-to-beat BPV (including SBP-CV and DBP-CV, respectively). The results showed that admission NIHSS score and SBP-CV were significant independent predictors of a poor in-hospital outcome in patients with spontaneous supratentorial ICH. SBP, Systolic Blood Pressure; DBP, Diastolic Blood Pressure; CV, Coefficient of Variation; NIHSS, National Institutes of Health Stroke Scale; ICH, intracerebral hemorrhage; BPV, Blood Pressure Variability.

Multivariate logistic regression analysis was performed to analyze the risk of poor in-hospital outcomes. The independent variables included in the analysis were admission NIHSS score, ICH volume, and beat-to-beat BPV (including SBP-CV and DBP-CV, respectively). The results showed that admission NIHSS score and SBP-CV were significant independent predictors of a poor in-hospital outcome in patients with spontaneous supratentorial ICH. The ORs, 95% CIs, and p-values are presented in Figure 1.

**Figure 2:**
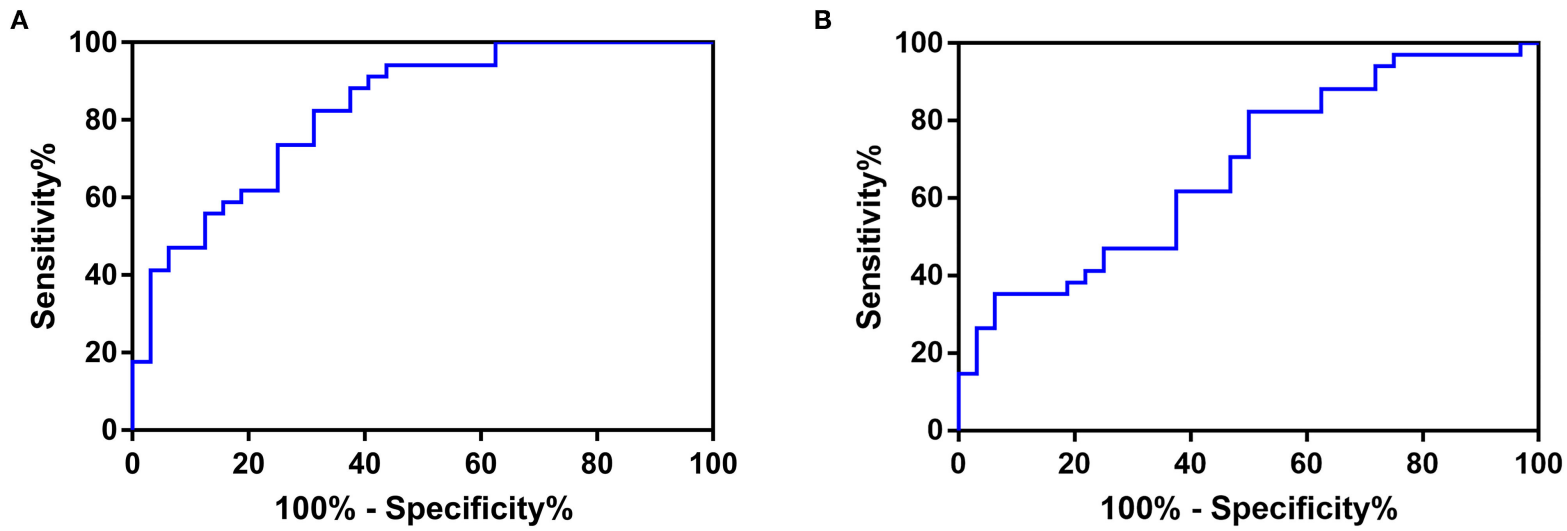
The ROC curve of **(A)** SBP-CV, and **(B)** DBP-CV in predicting poor in-hospital outcome in patients with acute spontaneous supratentorial ICH. The area under the ROC curve of SBP-CV for prediction of poor in-hospital outcome was 0.827 (95% CI, 0.730–0.925; *p* < 0.001), and the best cutoff point was 3.551 (sensitivity, 82.35%; specificity, 68.75%). The accuracy (Youden's index), positive predictive value and negative predictive value were 0.51, 73.68 and 78.57%, respectively. The area under the ROC curve of DBP-CV was 0.679 (95% CI, 0.551–0.808; *p* = 0.012), and the best cutoff point was 3.173 (sensitivity, 82.35%; specificity, 50%). The accuracy, positive predictive value and negative predictive value were 0.32, 63.63 and 72.73%, respectively. ROC, Receiver Operating Characteristic; SBP, Systolic Blood Pressure; DBP, Diastolic Blood Pressure; CV, Coefficient of Variation; ICH, intracerebral hemorrhage.

The ROC curve of the SBP-CV and DBP-CV is shown in Figure 2. The area under the ROC curve of SBP-CV for prediction of poor in-hospital outcome was 0.827 (95% CI, 0.730–0.925; *p* < 0.001), and the best cutoff point was 3.551 (sensitivity, 82.35%; specificity, 68.75%). The accuracy (Youden's index), positive predictive value and negative predictive value were 0.51, 73.68 and 78.57%, respectively. The area under the ROC curve of DBP-CV was 0.679 (95% CI, 0.551–0.808; *p* = 0.012), and the best cutoff point was 3.173 (sensitivity, 82.35%; specificity, 50%). The accuracy, positive predictive value and negative predictive value were 0.32, 63.63 and 72.73%, respectively.

## Discussion

This study investigated the effect of various parameters of beat-to-beat BP on the clinical outcome in patients with acute spontaneous supratentorial ICH. We demonstrated that SBP-CV and DBP-CV were significantly increased in ICH patients with poor outcomes. SBP-CV was an independent risk factor for a poor in-hospital outcome; the greater the variability, the poorer the outcome, with this relationship holding true even when admission and mean beat-to-beat BP levels were taken into consideration.

To the best of our knowledge, this is the first study to evaluate the association between beat-to-beat BP parameters and in-hospital outcomes and to compare the potential prognostic differences between the various parameters of beat-to-beat BP in patients with acute spontaneous supratentorial ICH. BPV is primarily divided into the three following categories: long-term (days to months), short-term (minutes to hours), and very short-term (beat-to-beat) BPV (Parati et al., 2013). Most previous studies in patients with ICH have focused on the first two types of BPV and have reported that an increased BPV during both acute and subacute stages was independently associated with worse functional outcomes at 3 months (Manning et al., 2014; Tanaka et al., 2014; Lattanzi et al., 2015; Chung et al., 2018; de Havenon et al., 2018; Meeks et al., 2019). Tanaka et al. and Rodriguez-Luna et al. further revealed that SBPV was correlated with early neurological deterioration (Rodriguez-Luna et al., 2013; Tanaka et al., 2014). The prognostic values of beat-to-beat BP parameters have recently been demonstrated in patients with ischemic stroke, and were confirmed to be more strongly associated with recurrent stroke and cardiovascular events than long-term and short-term BPV (Dawson et al., 2000; Webb et al., 2018). This indicates that beat-to-beat BP parameters may be a useful additional marker of cardiovascular risk. To date, only two studies have explored the changes in beat-to-beat BPV in patients with acute ICH. Sykora et al. demonstrated that baroreflex sensitivity was decreased in patients with acute ICH and that this decrease was associated with an increased beat-to-beat BPV. The authors also found that the 72-h beat-to-beat mean arterial pressure variability was significantly correlated with relative edema and early neurologic deterioration; however, this was only significant in a univariate analysis and was not duplicated in a stepwise multivariate linear regression analysis (Sykora et al., 2008, 2009). The present study showed a 2.5-fold increase in the risk of poor in-hospital outcomes for every unit of beat-to-beat BPV increase, independent of admission and mean beat-to-beat BP level. The beat-to-beat BPV results presented here are comparable to those previously reported after ICH when longer recording periods are taken. Divani et al. explored the association between BPV in the first 24 h of admission and in-hospital outcomes, and found that a higher SBPV was associated with unfavorable outcomes at discharge (Divani et al., 2019).

The indices of beat-to-beat BPV are diverse (Xia et al., 2017); considering the influence of the time sequence of individual BP measurements and mean BP levels on BPV, we chose SD, CV, ARV, and VIM as BPV evaluators in the present study. In the univariate analysis, only SBP-CV and DBP-CV were significantly higher in patients with unfavorable outcomes, although a trend toward significance was seen in other parameters. These results may indicate that CV is the most sensitive parameter for predicting short-term clinical outcomes in patients with ICH. CV was defined as the ratio of the SD and mean BP levels, and was correlated with mean BP, which suggests that the total variability and mean BP levels may interact to influence ICH outcome. The insignificance of VIM further supports this conjecture. In addition, CV does not take the time series variability into consideration, and no difference was found in ARV. This may suggest that the relationship between ICH outcome and BPV is not associated with the time sequence of individual BP measurements within only several minutes. That said, this finding might also be due to the relatively small sample size; further prospective studies are needed to further investigate this.

Although DBP-CV was associated with outcome in the univariate analysis, this association disappeared after adjusting for admission NIHSS score and ICH volume. In contrast, SBP-CV remained strongly associated with outcome. Moreover, SBP-CV predicted poor outcome better than DBP-CV according to ROC curves. Several previous studies have demonstrated that there is a stronger predictive function of short-term and long-term SBPV than DBPV in patients with ischemic or hemorrhagic stroke (Rothwell et al., 2010; Geeganage et al., 2011; Endo et al., 2013; Rodriguez-Luna et al., 2013; Chung et al., 2018; Divani et al., 2019; Meeks et al., 2019). This finding suggests that SBPV is more critical than DBPV for ICH prognosis, not only in short-term and long-term recordings, but also in beat-to-beat measurements.

The mechanism by which beat-to-beat BPV affects the outcome in patients with ICH is not fully understood. Increased beat-to-beat BPV reflects central autonomic dysfunction with sympathetic predominance and baroreflex impairment, which is associated with pro-inflammatory cytokine production, hyperglycemia, and increased blood-brain-barrier permeability (Raichle et al., 1975; van der Poll and Lowry, 1997; Watanabe et al., 2008; Jafari and Damani, 2020), all of which may worsen the outcome of patients with ICH. Dynamic cerebral autoregulation has been reported to be bilaterally impaired in patients with acute ICH, which is suggestive of an impaired ability to maintain a constant cerebral blood flow (Ma et al., 2016); this might also contribute to the worse outcome in patients with ICH who have a higher BPV. Sudden rises and falls in BP may promote hematoma enlargement and perihematomal ischemia, respectively (Menon et al., 2012; Rodriguez-Luna et al., 2013). Therefore, one potential therapeutic target is the stabilization of BPV during this vulnerable period, and this should be investigated in future clinical trials (Moullaali et al., 2019).

Some limitations should be considered when interpreting the findings. First, this study had a relatively small sample size, and this limited sample size meant that classes of antihypertensive medications were not selected as major covariates. Second, ambulatory BP monitoring data were not obtained in this study; hence, the different prognostic functions of short-term, long-term, and beat-to-beat BPV could not be compared. Finally, only hospital discharge outcomes were considered, and further prospective studies are warranted to identify any cause–effect relationship between beat-to-beat BPV and functional outcomes.

## Conclusion

In conclusion, a higher beat-to-beat BPV (SBP-CV) in the first 72 h of admission was associated with unfavorable in-hospital outcomes in patients with acute spontaneous supratentorial ICH. A greater variability was associated with a worse outcome, and this relationship remained true even when admission and mean BP levels were taken into consideration. The stabilization of BPV during the acute phase may be a therapeutic target for future clinical trials.

## Data Availability Statement

The raw data supporting the conclusions of this article will be made available by the authors, without undue reservation.

## Ethics Statement

The studies involving human participants were reviewed and approved by the Human and Research Ethics committees of the First Hospital of Jilin University. The patients/participants provided their written informed consent to participate in this study.

## Author Contributions

YY and Z-NG devised the study design and supervised study procedures. YQ, Z-NG, JL, and P-DZ analyzed the data and wrote the manuscript. HR, X-LY, and SQ collected the data. PZ provided significant statistical support. All authors provided critical review, edits, and approval of the final manuscript.

## Conflict of Interest

The authors declare that the research was conducted in the absence of any commercial or financial relationships that could be construed as a potential conflict of interest.
